# Peer Navigation to Support Transgender Women’s Engagement in HIV Care: Findings from the *Trans Amigas* Pilot Trial in São Paulo, Brazil

**DOI:** 10.1007/s10461-022-03595-8

**Published:** 2022-02-04

**Authors:** Sheri A. Lippman, Jae M. Sevelius, Gustavo Santa Roza Saggese, Hailey Gilmore, Katia Cristina Bassichetto, Daniel Dutra de Barros, Renata Batisteli de Oliveira, Luca Fasciolo Maschião, Dorothy Chen, Maria Amelia de Sousa Mascena Veras

**Affiliations:** 1 Division of Prevention Science, Department of Medicine, University of California, San Francisco, 550 16th Street, 3rd Floor, San Francisco, CA, USA; 2 Faculty of Medical Sciences, Santa Casa de São Paulo, São Paulo, SP, Brazil

**Keywords:** Gender affirmation, Behavioral intervention, Peer navigation, HIV care, Stigma, Transgender

## Abstract

Trans women living with HIV (TWH) have suboptimal HIV care engagement. We pilot tested Trans Amigas, a theory-based, trans-specific peer navigation (PN) intervention to address barriers to care in São Paulo, Brazil. TWH were randomized to the PN intervention (n = 75) or control (n = 38) condition. Control participants were referred to trans-friendly HIV care. Intervention participants were assigned a navigator who conducted nine in-person one-on-one sessions and bi-weekly phone or text check-ins to help participants overcome barriers to care and work towards gender affirmation and healthcare goals. We followed participants for 9 months to determine intervention feasibility, acceptability, and preliminary efficacy in improving retention in care. Analyses were intention to treat (ITT). Intervention acceptability was high: at end line, 85.2% of PN participants said they would continue receiving services and 94.4% would recommend peer navigation to a friend. A priori feasibility criteria were met: 92% of eligible participants enrolled and 70% were retained at 9 months; however, only 47% achieved moderate or better adherence to both in-person and phone/text program components. Though the pilot was not powered for efficacy, ITT findings trended toward significance, with intervention participants 40% more likely to be retained in care at the end of the study. Population-specific peer programming to support care engagement is acceptable, feasible, and can improve HIV outcomes for Trans women living with HIV.

## Resumen

Las mujeres transgénero que viven con VIH (MTV) tienen una participación subóptima en la atención del VIH. Nosotros evaluamos el programa piloto “Trans Amigas”, una intervención de navegación (o acompañamiento) de pares (NP) basada en un marco teórico, diseñado específicamente para mujeres transgénero, para abordar las dificultades de acceso a la salud en São Paulo, Brasil. MTV fueron asignadas de manera aleatoria a la intervención (n = 75) o a la condición de control (n = 38). Las participantes del grupo control fueron referidas a una unidad con servicios especializados para MTV. Las participantes en la intervención fueron asignadas a una navegadora, quien realizó nueve sesiones individuales en persona, así como llamadas telefónicas o mensajes de texto cada dos semanas para ayudar a las participantes a superar las barreras de acceso a la atención médica y avanzar hacia sus metas de afirmación de género y de salud. Seguimos a las participantes durante nueve meses para determinar factibilidad, aceptabilidad y eficacia preliminar en la retención en los servicios médicos. Usamos análisis por intención de tratar (AIT). La aceptabilidad de la intervención fue alta: 85.2% de las participantes NP dijeron que les gustaría seguir recibiendo los servicios NP, y 94.4% recomendarían NP a una amiga. El criterio de factibilidad a priori fue alcanzado: 92% de las participantes eligibles se inscribió y el 70% continuó por nueve meses. Sin embargo, solo el 47% alcanzó una adherencia moderada o alta tanto a las visitas en persona como a los mensajes de texto/llamadas telefónicas. Aunque el estudio piloto no tuvo el poder necesario para evaluar eficacia, el AIT reveló una tendencia hacia la significancia de que las participantes de la interventión tuvieran uma retentión en la atención médica un 40% mayor. Los programas NP que apoyan el la participatión en la atención del VIH, diseñados especificamente para la población, son aceptables, factibles, y pueden mejorar la situatión de salud de las mujeres transgénero que viven con VIH.

## Introduction

Transgender women (or ‘trans women,’ individuals assigned ‘male’ at birth but who identify as female or transgender) are at exceedingly high risk for HIV acquisition in Brazil [1], with disproportionate rates of infection, placing Brazil among countries with the greatest HIV disparities [2]. Prevalence among Brazilian transgender women has been estimated between 25 and 38% [3, 4], an odds of HIV infection over 55 times higher than the general population [5]. Consistent with data from the United States (US), where rates of HIV testing, antiretroviral therapy (ART) initiation, and viral suppression are significantly lower than other key populations [6–11], transgender women living with HIV (TWH) in Brazil also experience low rates of testing uptake [12] and viral suppression. One study in Rio de Janeiro estimated that only 35.4% of TWH had an undetectable viral load [13], as compared to an estimated 54% of adults living with HIV being virally suppressed nationally [14].

These marked disparities in HIV prevalence and treatment outcomes among TWH are due to complex socio-structural inequities that result in barriers to care access and engagement. Experienced discrimination from providers and concerns about potential interactions between ART and hormone therapy result in TWH avoiding care [15]. Further, social marginalization due to transphobia often results in poverty and unstable housing, familial alienation, limited formal education, mental illness, trauma and victimization, substance abuse, and introduction to sex work, often at an early age [4, 16–25], further limiting TWH’s access to care. Ethnographic and qualitative research in Brazil reaffirms high exposure to violence, substance use, and stigma and discrimination, which substantially limits access to public health and social services [25–27]. Findings from Projeto Muriel, a large survey of transgender populations in São Paulo, have indicated that 94% of trans women have experienced violence due to their gender identity [21], including 43% citing discrimination by health care providers [28]. Extreme discrimination and violence continues despite supportive legislation. Brazil has several progressive legal instruments supporting LGBT populations, including supreme court sanctioning of same-sex marriage, codified universal right to healthcare, and public coverage of gender-affirming surgery [29].

Despite grave need, few interventions exist to improve HIV care outcomes among TWH. To address this, we developed a trans-specific peer-based patient navigation (PN) intervention for the Brazilian context to address social, structural, and personal barriers to care. The intervention utilized elements of a successful peer-based program with trans women in the US [30, 31] and a PN study conducted in South Africa [32] with cisgender adults that improved retention in care [33, 34]. We examine feasibility and acceptability of the “Trans Amigas” intervention and explore initial efficacy among 113 TWH in São Paulo, Brazil who participated in a randomized controlled pilot trial to increase engagement in HIV care.

## Methods

### Study Procedures

The study was conducted in the São Paulo metropolitan area of Brazil in collaboration with the Faculdade de Ciências Médicas da Santa Casa de São Paulo. São Paulo, the largest city in Latin America, has a large transgender population and the highest concentration of gender affirming care services in Brazil [35]. Study procedures were conducted at Centro de Referência e Treinamento (CRT) DST/AIDS (São Paulo State HIV Reference and Training Center), home to a large outpatient clinic for HIV testing and treatment, and one of the first outpatient clinics for transgender health in the state of São Paulo.

From May to November 2018, TWH age 18 and over were recruited from the CRT testing and treatment center, through community-based outreach, and from among participants testing HIV-positive in the Transnational cohort [36]. The Transnational Cohort Study was a longitudinal cohort of transgender women recruited through respondent-driven sampling (RDS); 545 participants were followed for just over 2 years, including biannual surveys and HIV testing in order to establish incidence [37]. Participants testing positive or sero-converting in the Transnational study were referred to TransAmigas. To be eligible, participants had to be: 18 years or older; assigned ‘male’ at birth but currently identify as female, transgender, transsexual, or travesti (a common identity term used by trans women in Brazil); a resident of São Paulo; and have either a recent HIV diagnosis (prior 12 months) and be willing to enroll in HIV care at CRT or be enrolled at CRT for HIV care and willing to participate. Participants were enrolled following eligibility confirmation and informed consent.

Upon enrollment, participants responded to a survey and were then randomized at a 2:1 ratio to either the intervention (n = 75) or control (n = 38) condition; we chose to enroll more participants in the intervention group to ensure sufficient experiences with the navigation intervention to be able to evaluate acceptability and feasibility. All participants were followed over 9 months, with surveys at enrollment and 9 months, a brief telephone contact at 3 months to update contact information and check in, and ongoing extraction of clinical outcome data. Both surveys captured information about HIV care and treatment services; self-reported assessments of ART adherence; HIV risk behaviors and risk reduction practices; experiences of stigma, violence and transphobia; and measures of gender affirmation and other psychosocial covariates. The follow-up survey included an assessment of satisfaction with the intervention. Participants were reimbursed for travel at survey visits and provided with phone/data credits in the amount of R$20/month. Peer navigators (PNs) documented intervention delivery using contact forms, completed for each successful or attempted in-person meeting, phone call, or text message. Health outcomes were extracted from three data sources: (a) clinical visits from an electronic clinical charting system at the study clinic, (b) medication dispensing history from the national medications dispensing system (SICLOM), and (c) viral load (VL) results from the national laboratory tracking system (SISCEL). The study protocol was approved by the Committee for Human Research at UCSF, the Comitê de Ética em Pesquisa (CEP) at the CRT DST/AIDS—São Paulo, and the Brazilian National Ethics Committee, Conselho Nacional de Etica em Pesquisa (CONEP).

### Trans Amigas Intervention

Peer navigation is predicated on relationship building, support, and behavior modeling by peers who assess and help participants navigate barriers to engagement in HIV care and other services as well as improve ART adherence. The approach is rooted in social cognitive theory [38] and uses education and modeling from a similarly situated individual (i.e., the peer) to support and enhance a participant’s capacity and confidence to manage living with HIV and interface with health systems, including development of plans and strategies for overcoming barriers. Peer support has been shown to be a critical element of interventions with trans women [39]. In Trans Amigas, we incorporated the transgender-specific theoretical model of Gender Affirmation (GA) [10], which draws on objectification theory [40, 41], the identity threat model of stigma [42], and on research examining risk as an outcome of intersectional stigma [43, 44]. ‘Gender affirmation’ refers to an interpersonal process whereby a person receives social recognition and support for their gender identity and expression. The model of GA posits that in the context of transphobia, a high need for gender affirmation among TW, coupled with low access to gender affirmation, results in TW seeking to fulfill their unmet need in contexts that can pose health risks and result in diminished self-care. These health risks can include the use of industrial silicone and other unsafe fillers or engagement in unsafe sex, and can lead TW to prioritize gender-affirming aspects of health seeking behavior, such as hormone therapy, over HIV-related health behaviors such as adherence to treatment [10]. As a result, meeting trans women’s needs for gender affirmation, such as through gender-affirming relationships, health care, and peer-based support, can reduce risk behavior and increase self-care [45–47]. In Trans Amigas, we integrated gender-affirming programming into peer navigation through manualized curricula and exercises.

All PNs were TWH, receiving HIV care, living in the study area, and willing to share their HIV status with their participants. The initial multi-week PN training focused on: the navigation role (what it encompasses, what it does not); developing behavior change goals that are specific, measurable, attainable, realistic, and time-based (i.e., SMART goals); strategies for coping with transphobia in the context of health and social service facilities; fostering comfortable interpersonal dynamics; and ethical behavior in navigation and research, including appropriate personal/professional boundaries. Nine PNs assisted to up to ten participants each. PNs were supervised by a trained psychologist, with whom they met weekly to discuss the status of and challenges with participants as well as other research updates and personal challenges.

PNs contacted new participants within 2 weeks of enrollment. The initial intake visit included: (1) building rapport; (2) understanding current engagement in HIV care and ART adherence patterns; (3) identifying barriers to the participant’s engagement in HIV care and other health and social services; and (4) assisting in development of a dreams and goals plan. This plan included the participant’s personal goals for improving their overall health and their health as it related to HIV; their goals related to gender transition; and their goals for emotional well-being. The plan included notes on barriers to reaching these goals and steps to address those barriers, ideas on support needed to reach their goals, including how the PN could assist, and a plan of action with specific steps to achieve goals, which was revisited at each check-in. During follow-up visits, PNs worked with participants to identify specific changes they could make (or attempt) to overcome an identified barrier in the short- and mid-to long-term. PNs followed a standard session and check-in schedule, with 9 monthly in-person sessions, each with manualized content relevant to overcoming barriers to care, progress towards goals, gender affirmation, and communication skills to effectively navigate health care settings, and to problem solve barriers to care and wellbeing. In addition, PNs conducted at least one other check-in by text/WhatsApp or phone monthly. Check-ins focused on two topics: updates on attaining their goals and their planned actions as well as discussion about any new issues or challenges arising for the participants. PNs could make additional contacts, such as accompanying a participant to their social services or health appointments upon participant request. Over time, PNs encouraged participants to take increasing responsibility for identifying and implementing their own problem-solving strategies, so that they were ready to “graduate” from PN services after 9 months.

During the course of the project, PNs and their participants were also invited to participate in gender affirming and cohesion building group activities sponsored by the project. Group activities included “occupying public venues,” e.g. attending museums, art galleries, and social gatherings, as well as workshops, lectures, and a dance group that was coordinated by a physiotherapist with expertise in movement challenges resulting from injection silicone use. Workshops were conducted at the project’s community space, which was located in a building in central São Paulo that housed various community-based organizations dedicated to social programming.

The control condition consisted of direct referrals from study personnel to trans-friendly HIV care at the CRT clinic, the state reference center for HIV care and transgender health.

### Measures

Outcomes of the pilot study include intervention acceptability and feasibility, and preliminary efficacy of the intervention in impacting retention in care. Acceptability measures include proportion of TGW offered navigation services who accepted and started the program and proportion reporting satisfaction with navigation on the endline survey, including quality, duration, contact schedule, and the topics addressed during navigation. Feasibility measures include: enrollment rate, or the proportion of screened eligible participants who enrolled; proportion of TWH who successfully adhered to the program and completed navigation; and proportion of PNs who completed the research. An enrollment rate of ≥ 70% and a retention rate of 70% at 9 months was set as an a priori benchmark of feasibility [48]. Intervention adherence was assessed for in-person navigation and telephone or text-based navigation components utilizing data from participant contact forms. Full adherence was defined as completing eight or nine in-person sessions and at least 8 months of phone/text contact; moderate adherence included completion of four-seven in-person sessions and at least 4 months of phone/text contact; poor adherence included three or fewer in-person sessions and at least 1 month of phone/text contact.

Preliminary efficacy was assessed using medical records, national medication dispensing and laboratory exam systems. We defined retention in HIV care two ways—based on current literature. Being in care or retained at the end of the follow-up period was defined as having a confirmed HIV clinical visit or ART pick-up around 9 months (using the window of 7 ½–10 ½ months, given the 3-month treatment interval). We also assessed consistent retention, defined as having a least two visits or records of medication pick up, with no more than 6 months between visits during study follow-up. Finally, we explored defaulting from care, defined by the Brazilian Ministry of Health as absence from care or failure to access medication 90 days after prescribed medication is estimated to last [49]. There were too few participants who had no prior HIV care in the study population (n = 16) to assess linkage to care and only 48 participants with a viral load measurement near end-line, too few to assess viral suppression with sufficient precision.

### Analysis

Differences in participant characteristics by arm were assessed at baseline using chi-squared statistics for binary and categorical variables, t-tests for normally distributed continuous variables, and Kruskal–Wallis rank tests for non-parametric continuous variables. We analyzed measures of acceptability and feasibility among intervention participants using frequency tables. We also explored whether participant demographic characteristics were associated with feasibility (adherence to the program) and acceptability (would recommend the program) using the same comparative metrics.

Preliminary efficacy outcomes of retention and default were assessed using an intention to treat (ITT) approach, with multivariable logistic regression with robust standard error estimation comparing intervention to the control arm. Covariates, including housing status, age, partnership status, and hazardous drinking (assessed using the Audit-C) [50], were chosen a priori based on the literature. While the pilot was not powered to detect significant differences between the two arms, the point estimates of effect are intended to provide preliminary evidence of impact as well as early indicators of the sample size needed to power a future trial. Based on findings in our previous navigation trial [33, 34], we would have needed 328 participants to have sufficient power to detect an equivalent effect (OR 1.78) of the intervention on retention.

We included two kinds of sensitivity analyses. First, because clinical files were not located for 14.2% of participants, we repeated the ITT analysis using complete cases only. Further, to account for loss-to-follow up or censoring and to improve efficiency of estimates, we conducted sensitivity analyses using targeted maximum likelihood estimation (TMLE), a double-robust semi-parametric substitution estimator [51, 52]. TMLE utilizes a data-adaptive estimation technique to compute a causal estimate of the average treatment effect. We embedded the SuperLearner [53] prediction algorithm to improve model prediction in the presence of censoring.

## Results

**Fig. 1:**
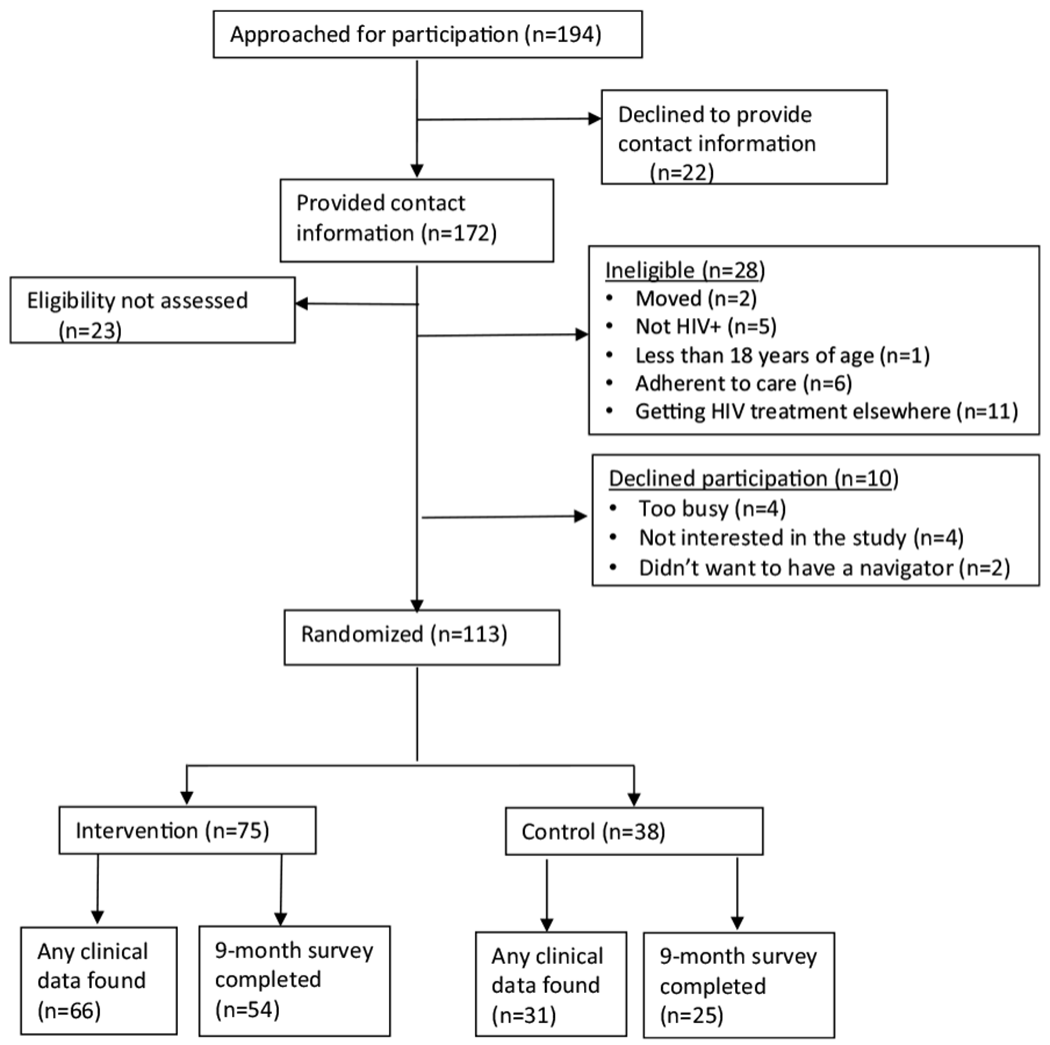
Trial population

A total of 194 potential participants were approached to assess interest and eligibility in participation. Of these, 172 provided contact information of which 149 (86.6%) presented for eligibility screening (Fig. 1). Among the 149 screened, 28 (18.8%) were found ineligible. Of those eligible, ten (8.1%) participants declined participation. We enrolled and randomized 113 participants, 75 (66.4%) in the intervention group and 38 (33.6%) in the control arm. Among those enrolled, 85.8% had clinical data extracted and 70% completed the endline survey. We found no statistical difference by arm in the proportion of participants with locatable clinical data and the proportion who completed the follow-up survey.

The mean age of participants was 32.9 years, 35.4% had completed a secondary education, and 30.1% were living at or under the poverty line (earning ≤ $800 Reais/mo ~ $220 US dollars), with 38.1% not consistently employed and 32.7% experiencing unstable housing (Table 1). Over 90% of participants had accessed primary care in the previous year, with just under 76% of the participants stating they were currently on ART. Demographic characteristics did not differ by arm.

### Acceptability and Feasibility

Acceptability was high, with all participants who underwent enrollment and randomization accepting the idea of peer navigation, though seven (9.3%) were never reached after enrollment (n = 3; disconnected phone or not found in contact locations) or never returned calls or texts (n = 4), resulting in 90.7% commencing navigation. Among those who responded to acceptability questions in the endline survey (54/68 receiving services), 85.2% said they would continue receiving services if possible, and 94.4% said they would recommend navigation services to a friend (Table 2). Overall, 50% of participants found the navigation very helpful, 33.3% found it somewhat helpful, and 16.7% found it not at all helpful. However, of the nine participants who found it not at all helpful, only two had actually met their PN in-person.

Both a priori feasibility criteria were met [48], with 92% of eligible participants enrolling and 70% retention at 9 months. However, only 30% achieved full adherence and completion of the navigation program, with 46.7% achieving moderate or better adherence to both in-person and phone/text contacts program components (Table 2). In some cases, in-person sessions became infeasible due to participant relocation, despite participants stating no intention to leave the area upon enrollment. Participants completed a median of six in-person meetings (IQR 2–9) and exchanged 86 calls or texts (IQR 27–118) with their PNs over the course of the intervention. Finally, of the nine PNs who began providing services, six (66.7%) completed the program, one left due to illness, one passed away, and one left due to difficulty with the job.

Because acceptability was very high, there was not sufficient variability in acceptability outcomes to identify demographic differences. However, some demographic characteristics were associated with adherence to the Trans Amigas program. Older age was associated with adherence to both calls/text check-ins (chi-sq = 4.54; p = 0.03) and to in-person sessions (chi-sq = 4.13; p = 0.04, Table 3). Additionally, those living in stable housing were more likely to adhere to calls and texts (chi-sq = 8.35; p < .01). While race/ethnicity was not significantly associated with adherence to the program at p < .05, those identifying as Black were less likely to adhere to calls/texts (chi-sq = 3.00; p = .08) and in-person sessions (chi-sq = 3.51; p = .06) when compared to those identifying as white or mixed race.

### Preliminary Efficacy

In ITT analyses assessing retention in care at the end of the study period, we found that participants in the intervention group were approximately 40% more likely to be in care at the end of the follow-up period as compared to control arm participants (RR 1.40; 95%CI 0.95–2.07; p = .07), and 28% (RR 1.28; 95%CI 0.95–1.70) more likely to have stayed in care continuously through the study, though neither finding reached significance at p ≤ .05 (Table 4). When restricting the data to complete cases in multivariable analyses, findings were attenuated slightly (RR 1.30; 95%CI 0.91–1.84) and (RR 1.19; 95%CI 0.95–1.51). Causal inference methods to account for censoring and covariates yielded similar results. Outcomes regarding defaulting from care were similarly favorable, with intervention participants 33% less likely to default (RR 0.67; 95%CI 0.27–1.57), though findings were not significant in this small sample.

## Discussion

We conducted a pilot trial of a theory-based peer navigation and support intervention for TWH designed to address social, structural, and personal barriers to care in São Paulo, Brazil. We found the intervention to be feasible and acceptable. While not powered to demonstrate efficacy, preliminary evidence showed that the intervention could impact retention in care and deter defaulting from treatment. Though almost all (94.4%) participants would recommend the intervention to others, only 30% fully adhered to the full program and only 47% moderately adhered to the program. Our findings demonstrate that trans women want and need targeted, efficacious programming that supports care engagement, but also face multiple challenges to sustained participation.

To our knowledge, this is the first pilot randomized trial of a PN intervention that demonstrates potential to impact retention in HIV care for TWH, including a possible 20–40% higher likelihood of remaining in care. Of note, this study was not powered for efficacy, resulting in differences in retention by arm that were not significant (p = .07). A few randomized trials have demonstrated efficacy of peer navigation in improving retention and viral suppression among populations that include trans women, but these did not report disaggregated data [54, 55]. A small number of quasi-experimental (pre-post design) studies similarly demonstrated that peer navigation improves HIV care engagement among TWH. For example, the Alexis Project [56], a combined peer health navigation and contingency management intervention with trans women of color in Hollywood, demonstrated that increasing attendance at navigation sessions was significantly associated with HIV visit attendance and viral suppression. Similarly, two demonstration projects in the San Francisco Bay Area [57] found that exposure to peer navigators was positively associated with retention in care among trans women of color. Intervention activities differed across studies, making it difficult to assess which aspects of navigation work best. However, all were theory-based, tailored to the specific location, and included true peers. We infer that theory-based, peer-based programming can improve HIV outcomes for populations experiencing structural inequities and intersectional stigma.

Many participants experienced barriers adhering to the navigation program, despite flexible meeting times and locations, maintaining contact during follow-up, and facilitating transportation. Under half of participants were able to attend four in-person sessions or more and maintain monthly communication for 4 months. Such findings are not new; studies have consistently found that the many structural inequities and challenges faced by transgender women, such as shortages of housing, money for transportation, or food or other basic needs, make it difficult to attend program and research visits [20, 58]. Adherence to the navigation program was particularly low among younger participants and among those without stable housing, suggesting multiple vulnerabilities. We also found some evidence that program adherence was lower among Black participants, a group subject to multiple forms of stigma and discrimination. Future efforts to address these structural barriers might include more focused work on housing, with PNs supporting participants in reaching goals around housing, identifying ways to stabilize housing situations, and partnering with housing-focused programs. Additionally, though a number of partnerships were in place, more extensive partnerships with community organizations could have provided more safe spaces or venues for navigation meetings, including evening venues. Additional qualitative research around barriers to participation with younger participants, who are more likely to be facing the complexities around their transition, and with Black participants could provide insights for better targeting intervention activities to their priorities, needs, and experiences. Finally, the advent of SARS-CoV2 has sparked proliferation of on-line support services, which might facilitate access to future programming. Navigation interventions could be conducted in the future with a combination of in-person and virtual visits, depending on ability to provide technical and financial assistance with phone or internet service and/or access to new platforms.

The Trans Amigas study has many strengths, including the randomized design and use of clinical visit and medication dispensing data to establish retention. The study’s primary limitation was small sample size; by design the trial was powered to assess acceptability and feasibility but not efficacy. Participants are not representative of all TWH in the area and were invited through partnering clinics and organizations, participant referrals, and past studies. That said, characteristics of our sample are similar to those of trans women in an RDS sample in São Paulo [3]. Finally, while extensive efforts were made to extract clinical case files and access national treatment and laboratory registers, we were unable to locate registry data for 14% of participants who may have been registered under a different name. Because randomization arm was not associated with missing data, missing information is likely to attenuate effects. Furthermore, to account for potential biases due to missing data, we conducted sensitivity analyses using the sub-set of complete cases as well as application of causal inference methods. Sensitivity analyses resulted in similar findings.

## Conclusions

This pilot trial of peer navigation to improve care engagement, informed by the Model of Gender Affirmation, was highly acceptable, feasible, and noted non-significant trends towards improved retention in care for intervention participants as compared to participants in the control arm. Future efforts should focus on approaches to reduce structural barriers to participation, including targeted research to understand means to facilitate access to programming for participants with the most difficulty engaging in services. Additional rigorous, longitudinal research with larger sample sizes is needed to corroborate findings and explore mechanisms of impact.

## Figures and Tables

**Table 1:** Sociodemographic, health-seeking, and behavioral characteristics of trans Amigas cohort by randomization group, São Paulo, Brazil

Respondent characteristics	Overall (n = 113)		Intervention (n = 75; 66.4%)		Control (n = 38; 33.6%)	
*Socio-demographic characteristics*						
Age (mean, SD)	32.9	10.2	32.1	10.0	34.6	10.6
Education						
Finished primary or less	41	36.3	25	61.0	16	39.0
Secondary incomplete	32	28.3	20	62.5	12	37.5
Completed secondary	40	35.4	30	75.0	10	25.0
Living in poverty (earned ≤ R$800/month)	34	30.1	27	79.4	7	20.6
Employment status						
Self-employed	51	45.1	36	70.6	15	29.4
Employed (with or w/out work card)	19	16.8	10	52.6	9	47.4
Student/retired/not consistently working	43	38.1	29	67.4	14	32.6
Partnership status						
Married/living together	42	37.2	32	76.2	10	23.8
Single	71	62.8	43	60.6	28	39.4
Self-identified skin color						
White	36	31.9	25	69.4	11	30.6
Black	13	11.5	7	53.9	6	46.2
Mixed race (parda)	54	47.8	37	68.5	17	31.5
Asian (amarela)	2	1.8	2	100.0	0	0.0
Indigenous	8	7.1	4	50.0	4	50.0
Living situation						
Stable housing	76	67.3	52	68.4	24	31.6
Unstable housing (SRO, staying with others, shelter, homeless)	37	32.7	23	63.2	14	36.8
Legal name on document?						
Yes	29	25.7	20	69.0	9	31.0
No	72	63.7	47	65.3	25	34.7
In process	12	10.6	8	66.7	4	33.3
*Access to health care*						
Accessed primary care in past year?	102	90.3	67	65.7	35	34.3
Accessed HIV care in past year?	92	90.2	60	65.2	32	34.8
Accessed gender transition care in past year?	26	25.5	19	73.1	7	26.9
ART status at baseline						
Yes—currently on ART	72	75.8	48	67.7	24	33.3
Had started but not on currently	18	18.9	13	72.2	5	27.8
Never on ART	5	5.3	3	60.0	2	40.0
*Behavioral characteristics*						
Number of sexual partners in the last 6 months						
0–1	28	24.8	20	71.4	8	28.6
2–9	31	27.4	17	54.8	14	45.2
10 or more	54	47.8	38	70.4	16	29.6
Gender of partners						
Cismen	112	99.1	74	66.1	38	33.9
Ciswomen	14	12.4	8	57.1	6	42.9
Transwomen or travestis	11	9.7	7	63.6	4	36.4
Transmen	6	5.3	3	50.0	3	50.0
Non-binary/gender non-conforming	4	3.5	2	50.0	2	50.0
Satisfaction with sex life						
Unsatisfied	16	14.2	8	50.0	8	50.0
Neutral	27	23.9	19	70.3	8	29.6
Satisfied	70	62.0	48	68.6	22	31.4
Alcohol use						
Hazardous alcohol use	59	52.21	40	67.80	19	32.20
No hazardous alcohol use	54	47.79	35	64.81	19	35.19

Overall column provides column percentages; Intervention and control columns present row percentages to facilitate assessment of differences in participant characteristics by randomization group

**Table 2:** Acceptability and Feasibility of peer navigation intervention among Trans Amigas intervention arm participants, n = 75

Acceptability—navigation participants	Overall (n = 75)		Endline Survey (n = 54)^£^	
	n	%	n	%
Proportion of TGW beginning navigation				
Yes—at least one telephone call/text	68	90.7	–	–
Yes—at least one in-person meeting	52	69.3	–	–
Never made contact	7	9.3	–	–
Would continue services if they could	–	–	46	85.2
Would recommend navigation to a friend	–	–	51	94.4
Helpfulness of navigation				
Very helpful	–	–	27	50.0
Somewhat helpful	–	–	18	33.3
Not at all helpful	–	–	9^¥^	16.7
Adequacy of face-to-face contact				
Not enough	–	–	29	53.7
Just about right	–	–	25	46.3
Too much	–	–	0	0.0
Adequacy of phone/text contact				
Not enough	–	–	13	24.1
Just about right	–	–	39	72.2
Too much	–	–	2	3.7
Feasibility—navigation participants				
In-person sessions completed				
Non adherent (no completed sessions)	23	30.7	–	–
Poor adherence (1–3 sessions)	17	22.6	–	–
Moderate adherence (4–7 sessions)	12	16.0	–	–
Fully adherent (8–9 sessions)*	23	30.7	–	–
Phone/text monthly communication				
Non adherent (no successful contact)	7	9.3	–	–
Poor adherence (1–3 months)	15	20.0	–	–
Moderate adherence (4–7 months)	26	34.7	–	–
Fully adherent (8–9 months)	27	36.0	–	–
Moderate or full adherence to both components	35	46.7	–	–
Total navigation minutes (median, IQR)	220.0	48.0–657.0	–	–

* Navigation completed; participant graduated

¥ 7 of 9 respondents never attended an in-person meeting

£ One endline respondent never made contact with their navigator

**Table 3:** Adherence to the program components by sociodemographic characteristics among 75 women randomized to intervention São Paulo, Brazil

	Adherence to telephone/text				Adherence to in-person navigation			
	Yes		No		Yes		No	
Moderately or fully adherent (n, %)	53	70.7	22	29.3	35	46.7	40	53.3
*Participant characteristics*								
Age (median, IQR)	31	25–40*	27	23–29	31	26-40^θ^	27.5	24–34
Education								
Finished primary or less	16	64.0	9	36.0	11	44.0	14	56.0
Secondary incomplete	13	65.0	7	35.0	8	40.0	12	60.0
Completed secondary	24	80.0	6	20.0	16	53.3	14	46.7
Living in poverty (earned ≤ R$800/month)	21	77.8	6	22.2	16	59.3	11	40.7
Employment status								
Employed (with or w/out work card)	7	70.0	3	30.0	4	40.0	6	60.0
Self-employed	28	77.8	8	22.2	16	44.4	20	55.6
Student/retired/not consistently working	18	62.1	11	37.9	15	51.7	14	48.3
Self-identified skin color								
White	19	76.0	6	24.0	13	52.0	12	48.0
Black	3	42.9^¥^	4	57.1	1	14.3	6	85.7^Ω^
Mixed race (parda)	27	73.0	10	27.0	19	51.3	18	48.7
Asian/Indigenous/other	4	66.7	2	33.3	2	33.3	4	66.7
Living situation								
Stable housing	42	80.8^£^	10	19.2	26	50.0	26	50.0
Unstable housing	11	47.8	12	52.2	9	39.1	14	60.9

Row percentages presented to facilitate assessment of differences in participant characteristics by adherence to program

Significant or marginally significant differences:

* chi-sq = 4.54, p = 0.03

θ chi-sq = 4.13, p = 0.04

¥ Black vs white and mixed race chi-sq = 3.00, p = .08

Ω Black vs white and mixed race, chi-sq = 3.51, p = .06

£ chi-sq = 8.35; p < .01

**Table 4:** Preliminary efficacy of the Trans Amigas peer navigation intervention, São Paulo, Brazil

	A. Retention in care at end of study period		B. Continuous retention in care (minimum 2 visits during study)		C. Default from treatment	
	RR*	CI	RR*	CI	RR*	CI
Intention to treat (n = 113)						
Control (ref)	–	–	–	–	–	–
Intervention	1.40	(0.95, 2.07)	1.28	(0.95, 1.70)	0.67	(0.27, 1.57)
Complete case analyses^θ^	n = 97		n = 99		n = 92	
Control (ref)	–	–	–	–	–	–
Intervention	1.30	(0.91, 1.84)	1.19	(0.95, 1.51)	0.63	(0.27, 1.50)
TMLE sensitivity analyses (n = 113)						
Control (ref)	–	–	–	–	–	–
Intervention	1.33	(0.96, 1.86)	1.20	(0.95, 1.51)	0.63	(0.27, 1.49)

* Multivariate logistic regression models controlled for partnership status, stable housing status, and alcohol misuse (TMLE model also included race/ethnicity, drug use)

θ Complete case samples vary due to different data sources (clinic visit data; laboratory and medications data; endline survey)

## Data Availability

The datasets generated during and analyzed during the current study are not publicly available, but are available from the corresponding author on reasonable request.
